# Neuromuscular Complications of SARS-CoV-2 and Other Viral Infections

**DOI:** 10.3389/fneur.2022.914411

**Published:** 2022-06-24

**Authors:** Sarah Jacob, Ronak Kapadia, Tyler Soule, Honglin Luo, Kerri L. Schellenberg, Renée N. Douville, Gerald Pfeffer

**Affiliations:** ^1^Hotchkiss Brain Institute, Department of Clinical Neurosciences, Cumming School of Medicine, University of Calgary, Calgary, AB, Canada; ^2^Centre for Heart and Lung Innovation, Department of Pathology and Laboratory Medicine, University of British Columbia, Vancouver, BC, Canada; ^3^Division of Neurology, Department of Medicine, University of Saskatchewan, Saskatoon, SK, Canada; ^4^Division of Neurodegenerative Disorders, Department of Biology, Albrechtsen St. Boniface Research Centre, University of Winnipeg, Winnipeg, MB, Canada; ^5^Department of Medical Genetics, Alberta Child Health Research Institute, University of Calgary, Calgary, AB, Canada

**Keywords:** COVID-19, SARS-CoV-2, neuromuscular disease (NMD), Guillain-Barre syndrome, viral disease, autoimmune disease

## Abstract

In this article we review complications to the peripheral nervous system that occur as a consequence of viral infections, with a special focus on complications of severe acute respiratory syndrome coronavirus-2 (SARS-CoV-2). We discuss neuromuscular complications in three broad categories; the direct consequences of viral infection, autoimmune neuromuscular disorders provoked by viral infections, and chronic neurodegenerative conditions which have been associated with viral infections. We also include discussion of neuromuscular disorders that are treated by immunomodulatory therapies, and how this affects patient susceptibility in the current context of the coronavirus disease 2019 (COVID-19) pandemic. COVID-19 is associated with direct consequences to the peripheral nervous system via presumed direct viral injury (dysgeusia/anosmia, myalgias/rhabdomyolysis, and potentially mononeuritis multiplex) and autoimmunity (Guillain Barré syndrome and variants). It has important implications for people receiving immunomodulatory therapies who may be at greater risk of severe outcomes from COVID-19. Thus far, chronic post-COVID syndromes (a.k.a: long COVID) also include possible involvement of the neuromuscular system. Whether we may observe neuromuscular degenerative conditions in the longer term will be an important question to monitor in future studies.

## Introduction

The emergence of the coronavirus disease 2019 (COVID-19) pandemic illness caused by severe acute respiratory syndrome coronavirus 2 (SARS-CoV-2) has led to the rapid publication of numerous reports outlining neuromuscular complications. Although many of the major complications of SARS-CoV-2 are related to the respiratory, cardiovascular, and host defense systems, the neuromuscular system can be affected in a large proportion of patients (1). In patients with critical illness or other comorbidities, neuromuscular complications may be difficult to recognize and thus be underdiagnosed (2).

Viral infections may affect the nervous system by a number of differing mechanisms. For the purposes of this review, we present three broad categories, (a) neuromuscular complications which are a direct consequence of viral infection (Table 1), (b) neuromuscular disorders which result from autoimmunity triggered by viral infection (Table 2), and (c) neuromuscular degenerative disorders which are associated with viral infections (Table 3) due to unclear mechanisms, or as part of a multifactorial etiology. This review discusses neuromuscular complications of viral infection broadly, with emphasis on SARS-CoV-2 because of its novelty.

**Table 1:** Viruses directly causing neuromuscular deficits.

**Virus**	**Neurologic complication/disorder**	**Observations**	**Mechanisms**
***Coronaviridae***			
Severe Acute Respiratory Syndrome Coronavirus 2 (SARS-CoV-2)	Parsonage-Turner Syndrome (PTS)	The association is uncertain or very rare. Isolated case reports have a temporal relationship to SARS-CoV-2 infection (3, 4).	No mechanisms have been proposed.
	Mononeuritis Multiplex (MNM)	Series of 11 patients with COVID-19 who required mechanical ventilation and ICU care (2).	The aetiology is unclear but authors proposed possibility of parainfectious vasculitis.
	Rhabdomyolysis	Multiple case reports of patients of rhabdomyolysis with SARS-CoV-2. Milder muscle involvement with myalgias and milder creatine kinase elevations appear to be very common (5).	Suggested mechanisms include direct invasion of myocytes by the virus, or immune mediated injury.
***Herpesviridae***			
Herpes simplex virus-1 or 2	Bell palsy	Lower motor neurone-type unilateral facial weakness.	Bell palsy remains of unclear aetiology but numerous lines of evidence suggest a role for HSV-1/2 and VZV in the infection of lower cranial nerves (see also VZV below) (6).
Herpes simplex virus-2 (HSV-2)	Lumbosacral plexitis (Elsberg syndrome)	Acute lumbosacral radiculitis which may also include lower spinal cord myelitis (7).	HSV-2 primary infection or reactivation.
Cytomegalovirus (CMV)	Mononeuritis Multiplex (MNM)	CMV reactivation in the context of AIDS due to HIV infection has been associated with MNM (8).	Autoimmunity is the proposed mechanism.
Varicella-Zoster Virus (VZV)	Post herpetic neuralgia	VZV reactivation (commonly known as shingles) can result in PHN in 10% of patients (9).	VZV remains dormant within dorsal root ganglia. Immune response to VZV reactivation is proposed to damage peripheral and central nervous systems leading to PHN.
	Lower Cranial Neuropathy (Ramsay Hunt syndrome)	Reports of cranial neuropathy associated with VZV reactivation (10).	Mechanism is unknown.
***Orthomyxoviridae***			
Influenza A and B virus	Influenza-associated myositis (IAM)	Influenza B is thought to be the major cause of viral myositis, mainly affecting calf muscles in males aged 5–9 with moderate CK elevations (11).	Postulated mechanisms include direct infection of myoctes or post-infectious immune mediated injury.
Human Immunodeficiency Virus (HIV)	Distal Symmetric Polyneuropathy (DSP)	A frequent complication of HIV presenting with numbness, tightness, burning pain, paraesthesiae, and allodynia (12).	The mechanisms are broadly suggested to be due to direct neurotoxicity of HIV, and as toxic consequences of antiretroviral therapy.
***Hepeviridae***			
Hepatitis E virus (HEV)	Parsonage Turner syndrome	Case reports suggest an association of acute HEV infection with PTS, which commonly has bilateral presentation (13).	Mechanism is unknown.
***Picornaviridae***			
Coxsackievirus	Myositis	A review of prior viral myositis and rhabdomyolysis cases showed coxsackieviruses as the second most common association (14) with broad age of onset from infancy to late adulthood.	
Echovirus	Myositis	Echovirus has been reported in cases predominnantly with young adult onset (14).	Direct viral invasion is suspected based on virus cultured from muscle specimen in one report (15).

**Table 2:** Viruses that have been linked to neuromuscular autoimmune disorders.

**Virus**	**Neurological disorders**	**Observations**	**Proposed mechanisms**
***Coronaviridae***			
Severe Acute Respiratory Syndrome Coronavirus 2 (SARS-CoV-2)	Guillain-Barré Syndrome (GBS)	Several case reports and series suggest a possible association (16). Larger epidemiologic studies have not confirmed the association. Atypical phenotypes such as facial diplegia may be more common.	GBS presentations have been described as para-infectious and post-infectious phenomena. The mechanism remains unclear but may be similar to mechanisms for GBS associated with other viral infections.
	Myasthenia Gravis (MG)	Multiple case reports of patients developing acetylcholine receptor (AChR) or muscle-specific kinase (MuSK) antibodies with generalized presentation, following SARS-CoV-2 infection (17).	Pathogenesis mechanisms of AChR-MG and MuSK-MG are distinct. May involve a break down in self-tolerance mechanisms.
***Orthomyxoviridae***			
Influenza A Virus	Guillain-Barré Syndrome (GBS)	A study showed that 26/150 (17%) GBS patients had a positive serology for Influenza A (18).	Molecular mimicry mechanism is a likely model in post-infectious GBS, however data that supports this hypothesis is sparse (19).
Influenza B Virus	Guillain-Barré Syndrome (GBS)	A study showed that 24/150 (16%) GBS patients had a positive serology for influenza B (18). 7 patients with preceding influenza B infection had a pure motor GBS without sensory deficits.	Molecular mimicry mechanism is a likely model in postinfectious GBS, however data that supports this hypothesis is sparse (19).
***Flaviviridae***			
West Nile Virus (WNV)	Myasthenia Gravis	Several months post WNV infection, MG was reported by 6 patients in one series (20).	Postulated to be from autoimmunity resulting in reduced self tolerance in initiating MG.
Zika Virus (ZIKV)	Guillain-Barré Syndrome (GBS)	A study in 2016 conducted during the Colombian outbreak of ZIKV showed 17/42 people (40%) tested for ZIKV were positive (21).	Parainfectious clinical presentation suggests a possible differing mechanism for ZIKV, still speculated to be from molecular mimicry or other immune dysregulation.
	Myasthenia Gravis (MG)	MG was presented in 2 case reports upon 8–10 weeks of ZIKV infection (22).	Unknown mechanisms, suspected environmental and genetic factors.
Hepatitis C virus (HCV)	Cryoglobulinaemic vasculitic neuropathy	Clinical presentation may include pure sensory polyneuropathy, sensorimotor polyneuropathy, or mononeuropathy multiplex. There appears to be female predilection (23).	Cold-insoluble immune complexes deposit on the vascular endothelium causing end-organ damage, including peripheral nerves (24).
***Phenuiviridae***			
Toscana Virus (TOSV)	Guillain-Barré Syndrome (GBS) - like syndrome	Report of a patient that was infected with TOSV which preceded GBS-like axonal polyneuropathy (25). Additionally, another study with 13 participants suggested a relationship to TOSV (26).	TOSV could be facilitating GBS immunological cascade. T-cell involvement and molecular mimicry mechanisms between axolemmal and microbial surface molecules could be considered.
***Picornaviridae***			
Enterovirus D68 (EV-D68)	Guillain-Barré Syndrome (GBS)	8 adult and 4 child cases of GBS, and variants of GBS such as AMAN, were reported in Wales (27).	Adult cases were all male. Geographic clustering of cases. This suggested combination of host genetic and environmental factors.
Hepatitis A Virus (HAV)	Guillain-Barré Syndrome (GBS)	A study showed that 7/150 (5%) GBS patients had a positive serology for hepatitis A (18). There was also a detailed study focused on a child with HAV infection that developed GBS (28).	Mechanisms unknown, may be a relationship to liver inflammation in addition to autoimmunity.
***Enteroviruses***			
Polymyositis	Autoimmune myositis	In adult study of 13 patients with myositis, 11 had idiopathic polymyositis (29). Study shows possible evidence of enteroviral infection being associated with autoimmune myositis.	Mechanisms unclear due to limited study.
Dermatomyositis	Autoimmune myositis	In adult study of 13 patients with myositis, 2 had dermatomyositis (29). Study shows possible evidence of enteroviral infection being associated with autoimmune myositis.	Mechanisms unclear due to limited study.
***Hepeviridae***			
Hepatitis E Virus (HEV)	Guillain-Barré Syndrome (GBS)	HEV RNA has been detected in cerebrospinal fluid (CSF) from some patients with HEV-associated GBS (30).	Two mechanisms have been proposed which include either direct viral damage or by molecular mimicry. HEV can infect neural cells in mouse models.
***Hantaviridae***			
Hantavirus (with chronic hepatitis B coinfection)	Chronic Inflammatory Demyelinating Polyneuropathy (CIDP)	Single case report in a HBV carrier with acute hantavirus infection (31). Nerve conduction studies (NCS) showed delayed latency in all 4 extremities. Polyclonal gammopathy with elevation of IgG and IgA.	MGUS is found in 10–20% of CIDP cases. The report suggested an interaction between the chronic HBV and hantavirus infection to induce CIDP with acute onset.
***Herpesviridae***			
Varicella-Zoster Virus (VZV)	Guillain-Barré Syndrome (GBS)	Patients were observed that had a VZV infection within 4 weeks of onset of weakness (32).	GBS may result from T-cell remodelling from VZV infection or ZVZ infection of peripheral nerves may provoke autoimmunity.
Epstein-Barr Virus (EBV)	Chronic Inflammatory Demyelinating Polyneuropathy (CIDP)	CIDP has been associated with elevated EBV IgG titres, and increased EBV copy numbers in circulating blood cells (33).	High viral loads and consequent immune responses may result in increased autoantigen recognition.
	Miller-Fisher Syndrome (MFS)	Individual case reports of MFS patients with anti-ganglioside antibodies (GQ1b) post EBV infection (34).	Possible molecular mimicry in which anti-GQ1b antibodies cross react with EBV surface antigens.
Cytomegalovirus (CMV)	Guillain-Barré Syndrome (GBS)	CMV IgM positivity is demonstrated in some cases of GBS. Severe clinical presentations are associated with anti-GM2 ganglioside antibodies following recent CMV infection (35).	IgM-type anti-GM2 antibodies are present in 30%-50% of GBS patients who have had recent CMV infection. The mechanism is unclear.
***Retroviridae***			
Human Immunodeficiency Virus (HIV)	Acute Inflammatory Demyelinating Polyneuropathy (AIDP) / Chronic Inflammatory Demyelinating Polyneuropathy (CIDP)	GBS/AIDP can occur during seroconversion from HIV infection, and prior to development of AIDS (12). Recurrences of AIDP and development of CIDP can develop in the months or years after seroconversion. AIDP and CIDP patients have been observed to have elevated CSF pleocytosis.	HIV is associated with coinfection with other viruses which may be the precipitant for autoimmunity, Or HIV may also specifically cause autoimmunity.
Human Endogenous Retrovirus-W (HERV-W)	Chronic Inflammatory Demyelinating Polyneuropathy (CIDP)	CIDP patients have increased MSRV-Env transcript levels (encoded by HERV-W), and is associated with inflammatory mediators that may be pathogenically relevant to CIDP (36).	CIDP autoimmune reaction may result from TLR4-driven activation of innate immunity by MSRV-Env, as shown in samples from human participants and human schwann cell cultures.

**Table 3:** Viruses possibly associated with chronic neuromuscular degenerative conditions.

**Virus**	**Neurologic disorder**	**Observations**	**Proposed mechanisms**
***Picornaviridae***			
Enteroviruses (EVs)	Amyotrophic Lateral Sclerosis (ALS)	EV genomic material was detected in spinal cord/brain of 60–88% of ALS patients compared 0–14% in controls (37). RT-PCR analysis of cerebrospinal fluid detected EV in 14.5% of 242 ALS patients and 7.6% present in 354 controls.	EV infection may lead to disease pathogenesis via seeding of protein misfolding and TDP-43 cytoplasmic aggregation.
Coxsackievirus B3 (CVB3)	Amyotrophic Lateral Sclerosis (ALS)	TDP-43 transactivation occurs during *in vitro* CVB3 infection (38). SODI-G58R mice infected with CVB3 have shortened lifespan with early onset and accelerated ALS-like motor dysfunction (39).	Cytoplasmic translocation and aggregation of TDP-43 is a hallmark for ALS, and CVB3 infection may contribute to this effect (40).
Echovirus-7 (echo-7)	Amyotrophic Lateral Sclerosis (ALS)	There were positive results from a neutralization test for echo-7 in over half (55%) of the ALS patients tested (41).	Exact mechanism is unclear. Echo-7 was explored because of the known ability of EVs to infect spinal and cortical motor neurons.
***Retroviridae***			
Human Immunodeficiency Virus (HIV)	Motor neuron disorder variations	Reports of HIV-positive patients having brachial amyotrophic diplegia (42).	Mechanism is unknown.
	ALS-like syndrome	HIV infection can be associated with ALS-like syndromes (43).	HIV is known to trigger the expression of HERV-K, which is associated with ALS neuropathology (44).
	Nemaline myopathy (NM)	In a study of 76 cases, HIV-NM cases showed similar presentation of features as those with sporadic late onset nemaline myopathy (SLONM) (45).	Formation of rods may be triggered by altered genome integrity, immunological triggers or direct impact of viral particles.
	Ocular myopathy	Reported patients with chronic progressive external ophthalmoplegia (CPEO) associated with long duration of HIV infection and antiretrovirals (46).	Prolonged HIV infection, or mitochondrial toxicity from therapy, or a combination of both may have resulted in these presentations.
	Sporadic Inclusion Body Myositis (sIBM)	Several reported cases of HIV-affected patients that developed IBM (47). An earlier onset age and higher CK level may be present compared to typical sIBM.	HIV-infected CD8+ T-cells may clonally expand within muscle tissues and cross-react with muscle surface antigens. Premature ageing and complications of antiretroviral therapy may be related.
Human T-lymphotropic Virus (HTLV-1/2)	ALS-like syndrome	In a study from 1995, 50% of sporadic ALS (sALS) patients showed immunoblot seroreactivity against HTLV-1/2 antigens (48). However, it is now recognized that HTLV can trigger ALS-like syndromes in some patients, and that the majority of patients with ALS are HTLV-1 seronegative (49).	HTLV has been associated with alterations in PTH regulation and motor neuron dysfunction. HTLV is also a known trigger of HERV-K expression, and thus may be associated with ALS-like neuropathology.
	Sporadic Inclusion Body Myositis (sIBM)	Reports of two patient that developed sIBM and tests and findings, such as anti-HTLV-1 antibodies in plasma and CSF suggest HTLV-1 was indeed present (50).	HTLV-1 infects mononuclear infiltrating cells that trigger IBM (51). It is also likely that retroviral infection and some sort inflammatory response also play a role.
Human Endogenous Retrovirus-K (HERV-K / ERVK)	Amyotrophic Lateral Sclerosis (ALS)	HERV-K *gag, pol*, and *env* gene transcripts are elevated in ALS brain tissues (52). Expression of HERV-K viral proteins is present in ALS pyramidal neurons and spinal cord oligodendrocytes (53).	HERV-K *env* protein can cause retraction and beading of neurites in human neurons. In transgenic mice, progressive motor dysfunction develops.

### Neuromuscular Disorders as a Direct Consequence of Viral Infection

Neuromuscular dysfunction may arise due to viral infections, from a variety of mechanisms which are not always well understood (Table 1). Viruses may directly infect cellular components of the central and peripheral nervous systems, causing injury in the process, or due to toxicity by viral proteins [ex.gp120 produced during HIV infection causing direct toxicity to neural cells (54)]. Neuronal retrograde viral dissemination occurs when infection of neurons occurs in the periphery and the virus co-opts cellular transport machinery to access the nervous system (55). Damage to the peripheral nervous system may also be a collateral injury from the innate immune response (56), although distinguishing direct viral injury from such collateral injury can be difficult. Certain viral infections (particularly herpesviruses) may remain dormant within neurons for years or decades (55).

Complications to muscle due to viral infection can occur indirectly as a downstream effect of peripheral nerve dysfunction, or as a direct effect of viral infection resulting in myositis (57). The precise mechanisms by which myositis develops are also unclear; certain viruses [paramyxoviruses, enteroviruses, and picornaviruses, among others (14, 58)] are known to be associated with myositis, presumably due to direct infection of muscle tissues (15). However, certain viruses are also thought to be involved in the pathogenesis of autoimmune myositis, without requiring direct or acute infection of muscle tissues (59).

It is also important to mention that severe systemic involvement due to viral infection may result in direct consequences to the neuromuscular system. When viruses result in critical illness and prolonged hospitalization, patients are at high risk of developing critical illness neuropathy and/or myopathy (60), and the longer-term consequences of muscle deconditioning and accelerated sarcopaenia (61). Organ dysfunction due to viral infection can also result in consequences to the nerves and/or muscles, such as when the renal (62), hepatic (63), or endocrine (64) systems become impaired.

### Neuromuscular Dysfunction Caused by Autoimmunity Triggered by Viral Infection

Viruses are an important cause of autoimmunity which can result in a wide spectrum of human diseases (65). Generally speaking, the mechanisms by which viral infection results in autoimmunity can include the following: (a) molecular mimicry, in which viral antigens similar to nervous system antigens provoke an autoimmune response to nervous tissues (66), (b) bystander activation, in which autoreactive T-cells respond to self antigens released from nonspecific tissue damage (67), and (c) epitope spreading, in which persistent infection results in progressive release of additional self antigens and diversification of self-epitopes that provoke an autoimmune response (68).

The classic neuromuscular complication from viral infection is Guillain Barré syndrome (GBS), either acute inflammatory demyelinating polyneuropathy (AIDP) or axonal variants thereof. This is also the group of conditions for which molecular mechanisms are best understood. Molecular mimicry was initially implicated in GBS based on studies of lipopolysaccharides from *Campylobacter jejuni* (*C jejuni*) that have molecular similarity to nerve sheath gangliosides (69). Further research demonstrated a group of antibodies autoreactive to gangliosides, associated with differing phenotypes of GBS (70). However, non-ganglioside antibodies have also been associated with GBS indicating a diversity of peripheral nerve proteins susceptible to autoimmunity due to presumed molecular mimicry (71).

Clinically, GBS is characterized by progressive and symmetrical weakness, with reduced myotatic reflexes (72). Approximately 50–70% of cases are preceded by respiratory or gastrointestinal infection within 1–2 weeks (73). *C jejuni*, influenza A, influenza B and hepatitis A serve as common preceding infection of GBS development, although there is substantial regional variation (18). Molecular mimicry is believed to be the primary mechanism for autoimmunity; *C. jejuni* has a ganglioside-like lipo-oligosaccharides (LOS) that accounts for the pathogenesis of axonal GBS (18). These axonal variants of AIDP, particularly acute motor axonal neuropathy (AMAN), may present with severe weakness and poor prognosis compared to demyelinating variants (32). However, AMAN may also be difficult to distinguish from AIDP and can have rapid reversibility of neurophysiologic findings in some cases, which can correlate to favorable prognosis (74, 75). A lengthy list of viruses is associated with GBS and these, as well as other autoimmune complications from viral infection are summarized in Table 2.

Chronic inflammatory demyelinating polyneuropathy (CIDP) results in relapsing disease which can be progressive and result in proximal or distal weakness (31). In its initial presentation, CIDP may present acutely, resembling acute inflammatory demyelinating polyneuropathy (or GBS) (31). CIDP may also occur following gastrointestinal or upper-respiratory infections (31).

Other conditions that have a possible autoimmune etiology include idiopathic brachial neuritis (also known as brachial amyotrophy or Parsonage Turner syndrome) characterized by pain and monoplegia (with occasional bilateral involvement). This condition is typically of unclear etiology, but has been associated with a wide range of possible underlying conditions, including various viral infections or vaccination (76). Post-infectious reactive myositis (also known as post-viral myositis) is typically described in children following influenza, and usually involves mild, self-limiting gastrocnemius weakness and pain (77).

### Neuromuscular Degenerative Conditions Associated with Viral Infection

Most of the discoveries connecting viral infections to chronic degenerative neuromuscular disorders is focused on ALS, a disease characterized by the degeneration of upper and lower motor neurons resulting in progressive paralysis (78). ALS is the most common motor neuron disease and is fatal, usually within 2–5 years of symptom onset (79). Half of patients (50%) diagnosed with ALS die within 30 months of symptom onset usually due to respiratory dysfunction, but 10% have been known to survive for more than a decade (79). Approximately 15% patients with ALS are diagnosed with frontotemporal dementia (FTD), and the two diseases can overlap pathologically and genetically (79). Based on European populations, up to 20% of patients with ALS were found to have a familial form (78). ALS incidence rates show regional variation for reasons that are not clear but could be related to environmental factors (78). There is no cure for ALS, nor has the pathogenesis been fully elucidated.

Neurodegenerative diseases like ALS can have a multifactorial component in their pathogenesis (80). The etiology of ALS may depend on several risk factors, including viruses. Viruses are considered environmental risk factors and therefore are also possible risk factors in ALS pathogenesis (79). Although precise causative mechanisms have not been defined, there are reported associations with several viral infections (Table 3), including enteroviruses and retroviruses.

## Discussion of Specific Viruses and Their Neuromuscular Complications

**Figure 1:**
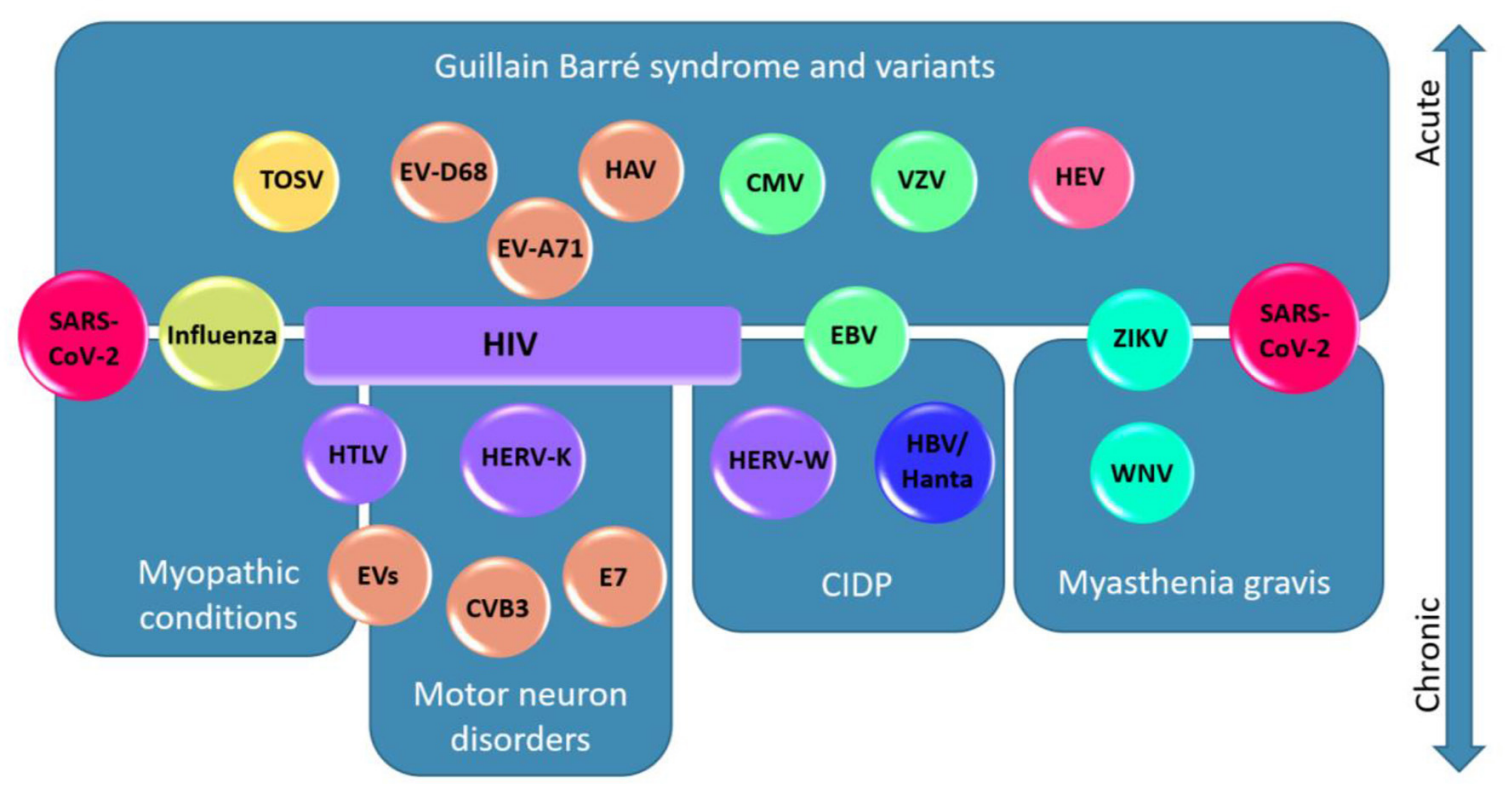
Viruses associated to their neuromuscular diseases and localisations. In this schematic diagram, viruses are represented according to the different diseases and neuromuscular localisations to which they are associated. Acute presentations are presented in the upper half of the figure and chronic disorders are presented in the lower half. Viruses are color-coded based on their family as follows: *Coronaviridae* (red), *Orthomyxoviridae* (yellow-green), *Flaviviridae* (cyan), Phenuiviridae (yellow), *Picornaviridae* (orange), *Hantaviridae* (blue), *Hepeviridae* (pink), *Herpesviridae* (green), and *Retroviridae* (purple). Note a general segregation of the viruses to the associated diseases/localisations. Note that SARS-CoV-2 is represented twice because of its association with GBS and variants, myasthenia gravis, and myopathic conditions. HIV is associated with a broad spectrum of neurological disorders and is therefore represented as a box spanning four disease categories. Abbreviations: Severe acute respiratory syndrome coronavirus 2 (SARS-CoV-2); Influenza A and B Virus (influenza); West Nile Virus (WNV); Zika Virus (ZIKV); Toscana virus (TOSV); Enterovirus (EV); Enterovirus D68 (EV-D68); Enterovirus A71 (EV-A71); Coxsackievirus B3 (CVB3); Echovirus-7 (E7); Hepatitis A Virus (HAV); Hepatitis E Virus (HEV); Hantavirus (Hanta); Hepatitis B virus (HBV); Varicella zoster virus (VZV); Epstein-Barr Virus (EBV); Cytomegalovirus (CMV); Human Immunodeficiency Virus (HIV), Human T-lymphotropic Virus (HTLV); Human Endogenous Retrovirus-W (HERV-W); Human Endogenous Retrovirus-K (HERV-K).

Both RNA and DNA viruses are known to elicit neuromuscular deficits. Many of these viruses share common pathological strategies to manipulate cellular function and promote neurological changes, such as viral protein neurotoxicity, induction of inflammation, autoimmunity, and modulation of cell signaling pathways (81–83). Below, viruses within select viral family groupings are discussed in detail as to their clinical presentation and association with neuromuscular disturbances. These associations are also schematically represented in Figure 1.

### Coronaviridae

#### SARS-CoV-2

##### Direct Neuromuscular Complications

**Figure 2:**
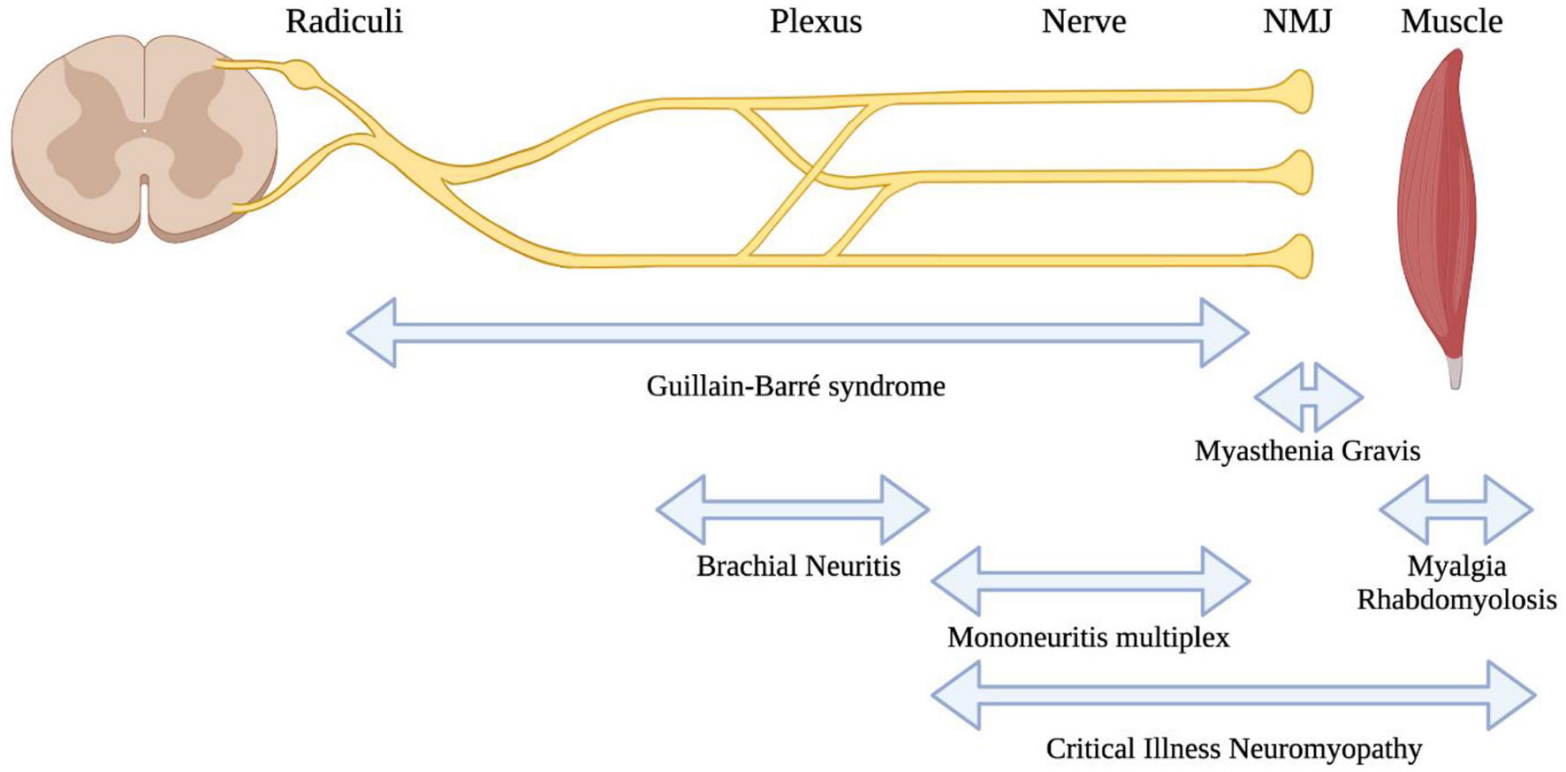
Localisations for neuromuscular consequences of SARS-CoV-2 infection. A highly schematic representation of the peripheral nervous system, showing major anatomic structures from the spinal cord through to muscle. Neuromuscular conditions associated with SARS-CoV-2 are listed below, with bidirectional arrows showing the approximate anatomic localisations of pathology.

SARS-CoV-2 is associated with direct involvement of both peripheral nerves and muscles (Figure 2). One peripheral nerve complication that is widely discussed in popular media is anosmia and dysgeusia. The prevailing theory regarding the mechanism for this relates to infection and inflammation in the olfactory epithelium, which highly expresses ACE2 [the receptor for SARS-CoV-2 infection (84)]. In contrast, olfactory neurones do not appear to express ACE2 (85), although it has been proposed that olfactory sensory neurones are the route of entry to the central nervous system (86). It is important to clarify that anosmia and dysgeusia are general complications from upper respiratory tract infections and the uniqueness of this clinical association with COVID-19 is still debated (87). Nonetheless, the anosmia and dysgeusia from SARS-CoV-2 infection are considered to be unique given the abrupt onset and recovery, and the molecular mechanisms for this are an area of continued investigation [recently reviewed (88)].

Muscle involvement in SARS-CoV-2 is well-described and appears to be very common in COVID-19 patients. This usually manifests as myalgias with elevations in creatine kinase enzyme levels (89). Severe muscle involvement with rhabdomyolysis is rarely associated with SARS-CoV-2, but the mechanism by which this occurs (direct viral infection or a consequence of the systemic inflammatory response) is not clear (5, 90). One study of muscle tissues from patients who died from COVID-19 pointed toward an immune-mediated pathogenesis for myopathy and cardiomyopathy, rather than direct infection of muscle tissues (91). This also appears to be supported by clinical presentations similar to dermatomyositis in some cases (92). Occasionally, rhabdomyolysis was the presenting clinical feature in advance of pulmonary symptoms from COVID-19 (93, 94).

Because SARS-CoV-2 may cause acute and severe illness requiring intensive care, these severe cases are also vulnerable to above-mentioned systemic complications including critical illness neuromyopathy, or downstream neuromuscular effects due to other end-organ injury or deconditioning. The use of dexamethasone for treatment of patients with severe disease results in overall better outcomes (95), although there is ongoing debate about whether corticosteroids may influence the development of critical illness neuromyopathy (96, 97). To our knowledge this has not been studied in the COVID-19 context.

##### Autoimmune Neuromuscular Complications

Guillain-Barré Syndrome (GBS) and its variants appear to be the most commonly described autoimmune neuromuscular complication from SARS-CoV-2 infection (98). The first reported case of GBS associated with SARS-CoV-2 infection appeared to suggest a parainfectious development of the condition (99). Since then, numerous additional reports have been published and recently reviewed (100). In general, GBS associated with COVID-19 occurs as a post-infectious condition, usually featuring classic clinical presentations of GBS. There is a broad age range of affected individuals and it can also occur in patients with asymptomatic infection (101). Phenotypic variations from the typical GBS presentations are also described after SARS-CoV-2 infection, such as isolated facial diplegia (102–104). Despite the many reports, a recent large epidemiologic study showed no association between COVID-19 and GBS; in fact, GBS incidence appeared to have been reduced during the pandemic, perhaps due to public health measures that have reduced the transmission of other infections (105). There are several reasons that an association may be challenging to detect, such as the difficulties in identifying people who could have had GBS following asymptomatic or paucisymptomatic COVID-19, and on the other end of the disease spectrum, identifying GBS in patients who expired or had prolonged critical care admission for severe COVID-19 (106). Future study will hopefully clarify if, or to what extent an association exists.

A single study demonstrated the finding of mononeuritis multiplex (MNM) in patients with severe SARS-CoV-2 infection (2) requiring critical care. Their nerve injuries were suspected to develop during the height of their illness, due to the recognition of their neuropathies following withdrawal of sedation. Proning and/or patient handling injuries were thought to be unlikely (2), although a separate study suggested that prone positioning may be associated with more frequent and atypical compressive neuropathies (107). The postulated mechanism was parainfectious vasculitis as part of the cytokine storm that has been observed from COVID-19 (108). This still awaits confirmation from independent study, although as the authors point out, MNM is very difficult to recognize in patients with critical illness, when findings of weakness are likely to be attributed to critical illness neuro/myopathy. We are aware of additional isolated cases of MNM that have been reported (109, 110).

Brachial neuritis is also described in association with SARS-CoV-2, in isolated case reports (3, 111, 112).

Overall, the recognition of neurologic complications of COVID-19 is highly important because they are associated with poor prognosis (113). There may yet be additional neuromuscular complications from COVID-19 which are unrecognized; for example, case reports suggest the possible association of COVID-19 in three patients with a new diagnosis of myasthenia gravis (17, 114). Further study is required to confirm whether associations from case reports are truly related to SARS-CoV-2 or are coincidental.

##### Chronic Post-COVID Syndrome (Long COVID) and Neuromuscular Complications

Infection from SARS-CoV-2 is increasingly recognized to produce long-term consequences in some patients, referred to by several names including the chronic post-COVID syndrome, post-acute sequelae of COVID-19 (PASC) syndrome, or popularly, “long COVID”. Symptoms that potentially implicate the neuromuscular system are common in long COVID, such as muscle weakness and fatigue, sensory disturbances, and symptoms of autonomic dysfunction (115). Small fiber neuropathy has been demonstrated in long COVID syndrome, using clinical and neurophysiologic evaluation (116) as well as pathologic confirmation (117). Myopathic changes are apparent using quantitative EMG in long COVID patients having fatigue symptoms (118). Autonomic dysfunction, primarily orthostatic intolerance, has also been shown with autonomic testing (119). This may also manifest as the postural orthostatic tachycardia syndrome (POTS) in some patients (120).

The mechanisms causing long COVID are unknown, but autoimmune processes or persistent uncleared viral material have been postulated, among others (121). Recently, evidence suggests that reactivation of Epstein-Barr virus, among other factors, appears to be associated with long COVID (122). Additional research will be required to understand this possible interaction and may begin to show added complexity to viral infections' impacts on human health.

##### COVID-19 and Patients with Pre-existing Neuromuscular Conditions

The COVID-19 pandemic has also brought attention to the added risk that patients with pre-existing neuromuscular disease may experience if they are exposed to SARS-CoV-2. This can be due to characteristics of the disease itself [e.g., causing pre-existing respiratory muscle weakness (123)]. Other aspects associated with having a neuromuscular disease may increase risk of exposure, such as carers entering the home and lack of accessibility to public health services (124). Several neuromuscular diseases are treated with immunosuppression which appears to be associated with higher risk of hospitalization and mortality due to SARS-CoV-2 (125).

Evidence suggests that patients with pre-existing neurological conditions have more severe outcomes from COVID-19 (113). Patients with neuromuscular disease may experience respiratory muscle weakness resulting in reduced airway clearance (123) or bulbar dysfunction with potential aspiration (126) which can result in higher risk of pulmonary infections, and lower resilience in the case of a comorbid respiratory infection such as SARS-CoV-2. Cardiac disease is also a common comorbidity with neuromuscular disorders that increases the likelihood of poor outcome from COVID-19 (127). Although specific evidence to show the extent of the added risk is still not available, there is clearly expert consensus that SARS-CoV-2 infection is likely to pose substantial added risk to neuromuscular patients (128). This has resulted in advocacy efforts for the prioritization of neuromuscular disease patients for vaccination (129). Patients with chronic disabling conditions may also be deprioritised for life-saving care in situations where critical care resources become limited (130, 131).

Living with a chronic neuromuscular disease also increases the risk of exposure to pandemic illness if caregiver support is required in the home (124). Patients with neuromuscular conditions may have reduced mobility and limited transport options, which may result in reduced access to public health services (132). Conversely, the pandemic has provided an opportunity to build virtual care programs to improve access and ease of care for patients with debilitating neuromuscular conditions. Telemedicine was an active area of research in certain neuromuscular conditions such as amyotrophic lateral sclerosis (ALS) even prior to the COVID pandemic (133), and patients/caregivers had identified the need for travel as the major barrier to ALS clinic attendance (134). Recent guidelines advocate for consideration of telehealth options for the care of ALS (135) and during the pandemic virtual care has been shown to be effective and well received (136).

We are aware of two reports suggesting that CIDP may be exacerbated by SARS-CoV-2 infection (137, 138). In both cases, the exacerbations were uncharacteristically severe in comparison to the patients' prior disease course: the former case had cranial nerve involvement and tetraparesis (137), and the latter having respiratory failure and cytoalbuminologic disassociation in CSF (138).

Autoimmune neuromuscular conditions such as myasthenia gravis are often treated with immune-suppressive medications. Evidence is conflicting as to whether immunosuppressive drugs increase susceptibility to severe outcomes due to COVID-19, although another recent cohort of immunosuppressed patients had higher likelihood of in-hospital complications and death (139). There is still no substantial evidence to address this issue, specifically for neuromuscular patients. Preliminarily, there is some suggestion that patients with myasthenia gravis may have more severe outcomes (140); however, it is challenging to determine the extent to which the disease itself or its therapies may contribute to this. In principle, there could also be concern that immune-suppression may reduce the efficacy of vaccination, although the limited evidence thus far suggests antibodies are detectable post-vaccination in immunocompromised patients, which is indicative of some degree of immunity (141). Evidence from other autoimmune disorders has led to general recommendations regarding the timing of vaccinations related to various immune-suppressive agents (142). Immune-suppressive agents can result in reduced antibody titres from vaccination (143), which appears to correlate with reduced immunity in some populations (144).

##### Vaccination Against SARS-CoV-2 and Neuromuscular Complications

An additional question of recent interest relates to whether vaccines against SARS-CoV-2 are associated with a heightened risk of GBS. There is controversy regarding whether vaccines in general, such as the seasonal influenza shot, may be associated with GBS (145). There have been a few case reports indicating GBS occurring in temporal relationship to SARS-CoV-2 vaccination (146–148), with general emphasis on the fact that current evidence does not support a causal link (146, 148). Some of the reported cases include patients who may have predisposing factors, such as a prior history of GBS following vaccination (149), although a cohort study of patients with prior GBS who received mRNA vaccination did not show any major complications and argues strongly against any major risk (150). Another report of two patients with remission from diffuse large B-cell lymphoma who developed GBS after vaccination with tozinameran suggesting that aberrant B-cell function may be relevant to risk of GBS (151). This remains an evolving area of research and recently there appears to be an association possible for the adenovirus-based SARS-CoV-2 vaccinations with GBS (152).

CIDP is also reported as a possible consequence of vaccination, again with the majority of cases associated with adenovirus-based products (153–155). Brachial neuritis is also described as a post-vaccination complication in several reports (156–158), including patients receiving mRNA vaccines and usually ipsilateral to the side of vaccination (159). Inflammatory myopathy has also been reported in isolated cases as a side effect of vaccination, causing syndromes resembling poly/dermatomyositis (160) or localized myositis near the site of inoculation (161), There are also case reports of SARS-CoV-2 vaccine associated with varicella zoster reactivation (162), although at present a relationship is not clearly established (163).

### Orthomyxoviridae

#### Influenza A and B Virus

The influenza pandemic (Spanish influenza), caused by a virulent strain of H1N1 of influenza A, affected 25–45% of the world's population, resulting in tens of millions of deaths (19). A study recruited 150 GBS patients and showed that 16% (24/150) of them displayed a positive serology for influenza B and 17% (26/150) had a positive serology for influenza A (18). Seven patients with preceding influenza B infection had a pure motor GBS without sensory deficits (18). This study (18) reported influenza B-related GBS as a pure motor phenotype. Molecular mimicry mechanism is a likely model in post-infectious GBS, however data that supports this hypothesis is sparse (19). Foreign and self-peptides share a common epitope; individuals carrying an MHC allele recognizing the target may activate autoreactive B cell or T cells through recognition of the foreign antigen, leading to autoimmunity (164).

According to one large retrospective study, 80% of cases of viral myositis were attributed to influenza, particularly influenza B (11). Influenza-associated myositis more commonly affects males aged 5–9, with calf muscle involvement and moderate elevations in creatine kinase. The condition is typically benign and resolves spontaneously, and it is likely to be under-recognized (165). Postulated mechanisms include the possibility of direct viral infection of myocytes (166), or perhaps as a post-infectious immune-mediated injury (167).

### Flaviviridae

#### West Nile Virus (WNV)

WNV is a positive-stranded RNA virus of the *Flaviridae* family (168). WNV can spread to humans by mosquitoes. Over 80% of WNV infections are asymptomatic, however approximately 5% symptomatic cases have neurologic manifestations including meningitis, encephalitis, and acute flaccid myelitis (168). There have been 6 reports of patients with WNV infection that led to myasthenia gravis, which may also suggest that WNV disease is a trigger for developing autoimmunity (20).

#### Zika Virus (ZIKV)

A study in 2016 conducted during the Colombian outbreak of ZIKV identified a series of 68 GBS patients with symptoms of ZIKV infection (21). Seventeen (25%) of these patients remained positive for ZIKV by RT-PCR, including 3 of the patients with ZIKV in the CSF, and was compatible with prior observations in a case-control study (169). Both studies suggested that the mechanism for GBS in these patients may differ from classic descriptions of GBS in other infections; the time to onset of GBS from viral infections was shorter and presence of viraemia at time of neuromuscular symptoms suggests a parainfectious, rather than postinfectious etiology (21). The association of GBS with ZIKV was also supported by a population study showing significant elevation in hospitalisations for inflammatory neuropathies at the same time ZIKV became prevalent in Brazil (170).

#### Hepatitis C Virus (HCV)

HCV causes chronic infection resulting in ongoing liver inflammation with long term complications including fibrosis, cirrhosis and hepatocellular carcinoma (171). Cryoglobulinaemic vasculitis occurs due to immune complexes that are cold-precipitating and deposit on the vascular endothelium of end-organs, such as peripheral nerves (24). Cryoglobulins are associated with chronic HCV infection but may also occur in lymphoproliferative disorders or other chronic conditions (172, 173). The peripheral nerve complications of cryoglobulinaemic vasculitis most commonly cause painful sensory or sensorimotor polyneurophathy, but also includes mononeuropathy multiplex (23). There appears to be a female predominance in development of peripheral nerve complications (23), and the central nervous system may also become affected (173).

### Phenuiviridae

#### Toscana Virus (TOSV)

TOSV originates in the Mediterranean area and is transmitted by sand flies (*Phlebotomus perniciosus* and *Phlebotomus perfiliew*i) in central Italy (174). While TOSV can affect the central nervous system (26, 174), TOSV IgG antibodies were found quite frequently in recently diagnosed GBS patients (26), suggesting that TOSV may trigger GBS. Similarly, a case report of a 40-year-old male had fit the diagnostic criteria for a subtype of GBS, acute motor and sensory axonal neuropathy (ASMAN) and tested positive for TOSV IgG antibodies as well (25).

### Picornaviridae

#### Enterovirus D68 (EV-D68)

This positive-strand RNA virus primarily shares characteristics with rhinoviruses and is primarily associated with respiratory disease, most notably during outbreaks starting in 2014 (175). During these outbreaks it was observed that EV-D68 was rarely associated with acute flaccid myelitis (176). There is a possible reported association of EV-D68 with GBS from one series (27).

#### Enterovirus A71 (EV-A71)

EV-A71 infection is a cause of the childhood exanthem referred to as “hand foot mouth disease”. A small proportion of cases are associated with central nervous system involvement (177), but a few reported cases have additionally associated this virus with presentations of Guillain Barré Syndrome (178, 179).

Isolated reports also support the possibility that enteroviruses can be associated with myositis. One study identified enterovirus-specific genomic sequences in autoimmune polymyositis and dermatomyositis (29). A single case report identified myositis and rhabdomyolysis coincident with echovirus-9 infection (180). A possible etiologic link is supported by animal studies that have connected other enteroviruses with myositis (181). However the association remains unclear with other studies unable to show viral persistence (182).

#### Hepatitis A Virus (HAV)

GBS is rarely associated with HAV (28) but there have still been reports across the literature. In the study by Hao et al. (18), 7/150 (5%) GBS patients had a positive serology for hepatitis A. There was also a case report of as 12-year old boy who met the diagnostic criteria for GBS upon HAV infection (28). In this latter report, it was unclear whether GBS was post-infectious, or occurring in the context of ongoing liver inflammation (28). HAV could be a rare trigger for GBS that may occur simultaneously with progressive liver inflammation (28). It is possible that select pathogenic mechanisms are shared by both HAV and GBS (28).

### Hepeviridae

#### Hepatitis E Virus (HEV)

The primary cause of hepatitis globally is HEV infection (30). There have been animal models where mice that were infected with HEV demonstrated the ability to infect neural tissue in the brain *in vivo* (30). HEV RNA has also been detected in CSF from some patients with HEV-associated GBS (30). Proposed mechanisms for GBS in HEV infection include either direct viral damage due to HEV replication in the neurological system and/or molecular mimicry (30). Although numerous case reports exist, only two prior studies report series of 10 or more cases (183, 184), suggesting HEV-associated GBS may be rare in endemic regions, or perhaps under-recognized and requires further study.

HEV has also rarely been associated with Parsonage Turner syndrome (PTS). Several of the reported cases have clear evidence of acute HEV infection (185–187). PTS associated with HEV appears to have a higher likelihood of bilateral presentation (13).

### Hantaviridae

#### Hantavirus

In a single case report (31), a 44-year old male who was a hepatitis B virus (HBV) carrier, presented with acute quadriplegia, fever, malaise, anorexia, and subconjunctival hemorrhage. His serology suggested acute hantavirus infection. Nerve conduction studies (NCS) showed delayed latency in all 4 extremities (31). The patient showed polyclonal gammopathy with elevation of both IgG and IgA (71). The patient was diagnosed with GBS, however due to relapsing disease, the diagnosis was felt to be more consistent with CIDP (31). HBV and hantavirus co-infection was proposed to induce CIDP (31).

### Herpesviridae

#### Herpes Simplex Virus 1 (HSV-1)

Bell palsy, which is the condition of unilateral paresis of cranial nerve VII, is a condition of unclear etiology. Certain Herpesviridae including HSV-1 (and HSV-2, below) are implicated in the pathogenesis of Bell palsy although various other causes are postulated (6).

#### Herpes Simplex Virus 2 (HSV-2)

HSV-2 infection is rarely associated with Elsberg syndrome, which is a presentation of acute lumbosacral plexitis, often accompanied by lower spinal cord myelitis (188). Elsberg syndrome may arise during either HSV-2 primary infection or during reactivation of latent infection (188). The condition may be under-recognized and may also be considered in unexplained cases of cauda equina syndrome (7). Criteria which may aid in recognition of this diagnostic entity have been proposed (7).

As mentioned above, HSV-2 is also considered to be associated with Bell palsy (6).

#### Varicella Zoster Virus (VZV, HHV-3)

VZV is also known as human alpha-herpesvirus 3 (HHV-3) and is most commonly associated with a febrile illness and macular-papular-vesicular rash during acute infection (varicella or “chicken pox”), as well as dermatomally-restricted eruptions during reactivation of dormant infection [zoster or “shingles (189)”]. Less commonly, varicella is associated with neurological complications which can include meningitis, encephalitis, stroke (due to vasculitis of cerebral vessels) and myelitis. Neuromuscular complications are common feature of VZV reactivation, in which patients may develop neuropathies of lower cranial nerves (10) [most commonly involving the facial nerve, referred to as Ramsay-Hunt syndrome (190, 191)]. VZV reactivation may also rarely resemble the clinical presentation of Elsberg syndrome (7). Post-herpetic neuralgia is a syndrome in which persistent neuropathic pain is present in the dermatome affected by a VZV reactivation (9). The spectrum of VZV reactivation syndromes may follow different patterns in the future as uptake of VZV vaccination continues (192).

GBS has also been associated with primary VZV infection. In one study, 1.6% of patients with varicella developed GBS within 4 weeks (32). It is speculated that VZV infection is associated with a pool of activated tissue-homing T-cells (32). CD4^+^ memory T cells are typically affected upon VZV infection (193). It is possible that this could lead to autoimmunity against peripheral nerves (32). However, it has also been suggested that VZV can directly infect the peripheral nerves which can result in peripheral nerve dysfunction (32).

#### Epstein-Barr Virus (EBV, HHV-4)

EBV is a highly prevalent DNA virus classically associated with mononucleosis, but is also associated with numerous human malignancies, and with non-malignant proliferative disorders in immune-compromised individuals (194). EBV is rarely associated with neuromuscular presentations including polyradiculitis [generally with coexistent central nervous system involvement (195)], as well as cranial neuropathy (196, 197), autonomic neuropathy (198), sensory neuropathy (199), and vasculitic neuropathy (200). These complications are generally only described in case reports and small series and proving their causation for such a widely prevalent infection is a challenge.

There may also be a connection between EBV infection and autoimmune neuromuscular disorders. Case reports connect EBV to GBS (201, 202) and variants such as Miller-Fisher syndrome (34, 203) and acute motor axonal neuropathy [AMAN (204)]. There is also a reported case of GBS with EBV and SARS-CoV-2 coinfection (205). There is also evidence suggesting that EBV is implicated in autoimmunity causing CIDP. A study including 66 CIDP participants showed that host-pathogen interactions were dysregulated for EBV, but not other herpesviruses, as evidenced by high seropositivity and threefold increased EBV cellular copy number compared with controls (33).

#### Cytomegalovirus (CMV, HHV-5)

CMV has high seroprevalence in the population, is well-adapted to the human immune system and typically does not produce symptoms, or occasionally results in mild illness resembling mononucleosis (206). Exceptions to this are in the case of congenital or neonatal infection, or in people who are immune-compromised due to HIV infection, organ or bone marrow transplants (207). In immunosuppressed adults, CMV is associated with the neurologic complication of polyradiculitis as an opportunistic infection (208).

Interestingly, immune-competent individuals with primary CMV infection may later develop GBS as a complication (209). A case-control study that enrolled 35 GBS cases found that in the 4 weeks preceding onset of GBS, 29 of the 35 patients experienced some form of infectious illness (35). Serologic testing identified 6 cases with IgM antibody against CMV, compared to 2 controls that showed the same result (35). Several other large studies confirm the strong association of primary CMV infection to GBS, including a 506 patient cohort study showing that CMV is a major cause of GBS comparable to *C jejuni* infection and present in 12.4% of cases (210). The pathogenesis may be explained by an association between anti-GM2 antibodies and previous CMV infection (211). IgM-type anti-GM2 antibodies, which are associated with motor neuron disease and acute and chronic polyneuropathies, are present in 30–50% of GBS patients who have had CMV infection (211).

### Retroviridae

#### Human Immunodeficiency Virus (HIV)

Neurological diseases manifest during all stages of HIV infection, affecting the central and/or peripheral nervous systems (12). Neuromuscular complications from HIV may arise as a direct consequence of HIV infection, but can also be secondary to the acquired immunodeficiency syndrome (AIDS) (212), or as a consequence of antiretroviral therapy (ART) (213). HIV has been associated with complications at almost every possible neuromuscular localization, including lower motor neurons, polyradiculitis, polyneuropathy (including GBS and variants), autonomic neuropathy, mononeuropathy, mononeuritis multiplex, and myopathies (sporadic inclusion body myositis and sporadic late onset nemaline myopathy) (12, 42, 214).

Distal Symmetric Polyneuropathy (DSP) is the most common neurological complication resulting from HIV, affecting over 30% of HIV patients (215). DSP presents with symptoms including numbness, tightness, burning pain, paraesthesiae, and allodynia. The likelihood of developing DSP correlates with increased height, diabetes, substance abuse, hypertriglyceridemia, metabolic syndrome, increased age, and exposure to antiretroviral medications (12). The pathogenesis of this common complication may be related to dysregulation of macrophages and release of pro-inflammatory cytokines (TNF-α, IFN- α, IL-6), nitric oxide dysregulation, and free radical damage (12). Direct toxicity from the gp120 protein and an interaction with antiretrovirals may also be relevant to pathogenesis (216).

GBS or AIDP can rarely manifest in the initial stages (seroconversion) of HIV infection (12). Patients may present with symmetric acute ascending motor weakness, loss of reflexes, sensory symptoms and these symptoms usually precede with a flu or diarrheal illness (12). HIV patients with AIDP may have frequent episodes of AIDP and/or develop CIDP (12). Both AIDP and CIDP show elevated pleocytosis, meaning it is likely related to cerebrospinal fluid HIV viraemia (12). The immune response results in demyelinating the myelin sheath via autoantibodies and can affect multiple areas of the peripheral nervous system (12).

Mononeuritis multiplex (MNM) has been associated with HIV, in situations where AIDS occurs in conjunction with cytomegalovirus (CMV) reactivation (8). MNM is therefore unlikely to be present in patients who have access to ART. CMV reactivation can present with multi-organ involvement including retinitis, colitis, and oesophagitis (8). More extensive neurological involvement may also occur, manifesting as encephalitis, myelitis, and/or polyradiculopathy (8). Sensory ganglionopathy is also a possible presentation of HIV that has rarely also been associated with other viral etiologies (217).

#### Human Endogenous Retrovirus-W (HERV-W)

HERV-W encodes a pro-inflammatory protein named Multiple Sclerosis-associated RetroViral element Envelop protein (MSRV-Env) (36). One study investigated inflammatory neurological diseases for a study on Multiple Sclerosis and observed MSRV-Env expression (36). The study had 2 cohorts, part of which included 31 CIDP patients (36). In Study 1, 7/15 CIDP patients had significantly elevated levels, also known as a High Expression profile (HE), for MSRV transcript expression, majority (6/7) had a HE for MSRV-Env, and 1 had a HE for MSRV-pol (36). In Study 2, 6 CIDP patients presented with a HE for MSRV-Env and 8 CIDP patients presented a HE for MSRV-pol (36). There was also elevated Interleukin 6 (IL-6) and C-X-C Motif Chemokine Ligand 10 (CXCL10) levels in CIDP serum (36). Upregulation of IL-6 and CXCL10 is induced by MSRV-Env (36), which is consistent with the study's findings (165). CIDP autoimmune reaction may result from TLR4-driven activation of innate immunity by MSRV-Env (36). As observed in human Schwann cells (HSC), MSRV-Env expression within CIDP peripheral nerves may trigger inflammation along peripheral nerves mirrored by systemic immune dysregulation (36).

## Discussion of Specific Viruses and Neuromuscular Degenerative Conditions

### Picornaviridae

The picornavirus family has been investigated as a possible aetiologic agent in the development of ALS, given the presence of picornavirus RNA in central nervous system that is predominant in the anterior horns of the spinal cord (218).

### Enteroviruses and ALS

Enteroviruses (EVs), including poliovirus, EV-D68, EV-A71, coxsackievirus B3 (CVB3), and echovirus-7 (Echo-7), tend to target several cell types in the CNS. They are able to infect motor neurons and cause the development of post-poliomyelitis-syndrome or non-polio flaccid paralysis (37). EV-D68 and EV-A71 are typically responsible for epidemics across the North American region and Asia-Pacific, respectively (219, 220). EV genomic material was detected in 60–88% of spinal cord/brain from ALS patients compared to 0–14% from controls (37). RT-PCR analysis of cerebrospinal fluid also showed detection of EV in 14.5% of 242 ALS patients vs. 7.6% in 354 controls, suggesting a link between EV infection and ALS development (37). EV infection is plausible to cause disease pathology via seeding of protein misfolding (37). An active viral infection is not necessarily required for disease progression because if EV infection occurs during this acute phase in childhood, it may still gradually propagate misfolded proteins in other regions of the CNS, eventually leading to ALS onset in adulthood (37).

Intriguingly, recent studies on CVB3 revealed that viral infection produces hallmark molecular and pathological phenotypes of ALS both *in vitro* and *in vivo* (38–40). CVB3 infection has been shown to cause translocation of TDP-43 to the cytoplasm, a pattern seen in other neurodegenerative diseases, through the action of virus-encoded protease that disrupts the nuclear pore complexes. CVB3 infection also results in the formation of stress granules (SGs), which co-localize with cytosolic TDP-43 aggregates (40). Cytoplasmic translocation and aggregation of TDP-43 is a hallmark for ALS (40, 41). In addition, CVB3 infection can cause direct neuronal damage and trigger robust inflammatory responses. Animal studies using SOD1-G85R ALS mice demonstrated that sublethal CVB3 infection that mimics chronic infection leads to early onset and accelerated ALS-like motor dysfunction, and shortened lifespan (39).

Echoviruses are part of the Enterovirus genus (41). In one study, they had positive results from a neutralization test for echo-7 of 40% among referents and 55% in ALS patients (221). The study chose to explore echo-7 because of the known ability of EVs to infect and destroy spinal and cortical motorneurons (41, 221). However the potential link to echoviruses should be studied further, with one prior study showing absence of echovirus RNA in CNS tissues of ALS patients (222).

### Retroviridae

#### HIV and ALS-Like Syndromes

HIV appears to be capable of injuring the neuromuscular system with varying clinical presentations, localisations, and pathologic features. There have been over 20 case reports of HIV-associated motor neuron disease (12). Both HIV and human T-cell leukemia virus-1 (HTLV-1) have been associated with syndromes resembling ALS and respond to antiretroviral therapy (223, 224). Clinical observations have reported some ALS-like similarities and asymmetric patterns of extremity weakness, upper and lower motor neuron signs, fasciculations, atrophy, weight loss, easy fatigability, and brisk muscle jerk reflexes (12). HIV has also rarely been associated with motor neuron dysfunction causing bibrachial amyotrophic diplegia in case reports (42). HIV may lead to motor neuron disease by direct or indirect mechanisms of the virus (12). The virus can infiltrate the macrophages, microglia, and astrocytes, which affects neurons in the CNS (12).

Late-onset myopathy has also been associated with HIV infection, including cases of ocular myopathy presenting with ophthalmoplegia and/or ptosis (46, 225). Mitochondrial dyfunction was suggested as the underlying pathogenesis, based on the resemblance of this clinical presentation to chronic progressive external ophthalmoplegia (CPEO) (226). However, whether this relates to mitochondrial toxicity from HIV infection itself, and/or antiviral therapies is not known. Some of the reported cases had large-scale mtDNA deletions (46), so it is uncertain whether HIV and therapy unmasked or accelerated pre-existing disease, or was simply coincidental. Other rarely described late-onset myopathy presentations with HIV include sporadic inclusion body myositis (sIBM) and sporadic late-onset nemaline myopathy (SLONM) (42, 50). Described cases of sIBM suggest the age of onset may be earlier and with higher serum CK than HIV-negative sIBM (47). In the case of SLONM associated with HIV, cases appear to respond to immunosuppression emphasizing the importance of obtaining HIV serology in the workup of SLONM (45).

#### Human T-Lymphotropic Virus and ALS-Like Syndromes

In a study from 1995, 25 out of 50 sporadic ALS (sALS) patients showed immunoblot seroreactivity against HTLV-1/2 antigens (48). However, it is now recognized that most patients with sALS are HTLV-1 seronegative, although some HTLV-1 patients may have clinical presentations resembling motor neurone disease (49, 227), which probably is the reason for the finding in the above study. Mechanistically, HTLV has been associated with alterations in parathyroid hormone (PTH) regulation, which can result in motor neuron dysfunction (48). HTLV may also activate various human endogenous retroviruses (228) which may be implicated in neurodegenerative disease (see discussion below). Motor neuron disease can also be mimicked by lymphoma (a potential consequence of HTLV-1 infection) (48).

Sporadic inclusion body myositis has also been described from case reports in patients with evidence of HTLV-1 infection (50, 51). A series of patients from an endemic area in Japan appears to support sIBM as a true but uncommon association with chronic HTLV-1 infection (229).

#### Human Endogenous Retrovirus-K (HERV-K/ERVK) and ALS

Human endogenous retrovirus-K (HERV-K/ERVK) is a genomic symbiont in primates. Specifically, the HERV-K subgroup human mouse mammary tumor virus-like 2 (HML-2) is of particular interest because it is a recent integrant into the human genome and can become reactivated in several disease state, such as neurological disorders, autoimmune disorders and cancer (230).

For over a decade, HERV-K has been shown to have a neuropathological association with ALS (52). The reactivation of HERV-K is associated with upregulation of the RNA binding protein TDP-43, which is a defining neuropathologic feature in most cases of ALS (52, 53). This association has potential implications for biomarker development and therapy, and there are reports indirectly suggesting response to antiretroviral therapy in some ALS patients (223, 224, 231); further studies to confirm clinical significance will be required.

In comparison to healthy patients, HERV-K has been shown to have a significant elevated expression of its *gag, pol*, and *env* gene transcripts in the brain tissue of ALS patients (53). However, this is an evolving research area and recent studies indicate that experimentally assessing the relationship between HERV-K transcript levels in ALS may not be straightforward (232, 233). In contrast, the expression of HERV-K in brain and spinal cord is reliably measured at the protein level in approximately half of patients with sporadic ALS (52, 53, 224). A variety of HERV-K viral proteins can be measured at the protein level, including the viral envelope protein, conotoxin-like protein, reverse transcriptase, integrase and structural Gag proteins in specimens from patients with ALS. Moreover, a transgenic murine model of HERV-K driven motor neuron disease highlights that the production of viral proteins from the HERV-K *env* gene in neurons can lead to motor disturbances and reduced lifespan (53). It will be important for future studies to investigate how the neuropathological mechanisms behind HERV-K's association with ALS differ from that of other viruses, and whether appropriate antiviral drugs may be used therapeutically to treat motor disturbances in ALS.

Increased HERV-K protein maturation has also been observed in Spinal Bulbar Muscular Atrophy, which is an inherited form of motor neuron disease (234).

## Discussion

This review presents a summary of neuromuscular complications associated with viral infections. The complications associated with SARS-CoV-2 infection have been emphasized because of its novelty. Of greatest interest is the possible relationship of GBS and variants to SARS-CoV-2. Anecdotally there are numerous reports of GBS in the literature temporally related to COVID-19, and it appears possible that atypical presentations such as facial diplegia may be more commonly associated with SARS-CoV-2. However, large epidemiologic studies have not clearly demonstrated an association, and paradoxically the incidence of GBS even appears to have been reduced by the pandemic (105). This reduction is likely because public health restrictions have been highly effective at reducing other infections that are strongly associated with GBS, such as *C. jejuni* and various respiratory viruses. A lack of association between SARS-CoV-2 and GBS is considered reasonable because SARS-CoV-2 epitopes are not known to have homology to tissues of the neuromuscular system (105).

Because SARS-CoV-2 is a novel illness, various unanswered questions remain. At time of writing there were new genetic variants with higher virulence that were increasing in prevalence (235). Whether this will affect the prevalence of direct or autoimmune neurologic complications from SARS-CoV-2 infection is unknown but vigilance by clinicians and health systems will be necessary.

As seen in Table 3, many viruses are associated with the longer-term development of neurodegenerative conditions. It is certainly too early to know whether any neurodegenerative long-term complications of COVID-19 exist, but it will be necessary to remain alert to this possibility. Already there is some evidence that SARS-CoV-2 has long-term effects on disparate organ systems (115), which is a general phenomenon also seen with other viral infections (236). The most common long-term symptoms (chronic post COVID syndrome, also known as “long COVID”) include muscle weakness and fatigue (115), although whether this is due to effects on the neuromuscular system, other organs, or other factors, is still unknown. Overall, the persistence of long-term symptoms appears to be associated with severe disease during acute infection requiring hospitalization (237, 238). In such patients, critical illness neuromyopathy may be present to varying degrees as a general consequence of critical care management (239). However, long-term symptoms are also described in patients without severe disease and understanding the full extent of this syndrome and its mechanisms will require extensive further study (240).

An important issue occurring in the context of the SARS-CoV-2 pandemic is the relationship of persons receiving immunosuppressive therapies and their risk of poor outcomes from COVID-19. As discussed in our article, several neuromuscular disorders are treated with immunosuppression (241, 242). Since such therapies can be associated with opportunistic infections and poor outcomes from other infectious agents (243) there was concern that severe outcomes would also occur from SARS-CoV-2. This question probably remains unanswered; neuromuscular patients were strongly encouraged to limit their risk of exposure (244), so case counts in neuromuscular patients may have remained disproportionately low as a result. Another possibility is that immunosuppression may not be a risk factor, or may even be protective, as has been suggested in systematic analysis (245).

Overall, the range of possible neuromuscular complications relating to viral infections is broad. Some viruses such as HIV have been broadly studied and have an array of possible consequences to the nervous system; others have been associated only with restricted phenotypes and may be limited solely to anecdotal observations. Our knowledge of neuromuscular or other organ system complications from viral infections is likely to be incomplete due to the challenges with connecting both common and uncommon infections with rare neuromuscular disorders. The interaction of viral infections with neuromuscular disorders is likely to be complex and influenced by environmental factors, host genetic factors, as well as the microbiome (246), among other factors that may not yet be discovered. The underlying molecular mechanisms are likely to be diverse and with complex inter-relationships. Future research in these areas will provide a better understanding of the pathogenesis of neuromuscular disorders and hopefully open new therapeutic targets.

## Author Contributions

SJ was the primary author and performed the literature review. RK, TS, HL, KS, and RD revised the manuscript for intellectual content. TS, RD, and GP conceptualized and generated the Figures. GP authored the manuscript, conceptualized the project, and supervised the work. All authors approved the final version submitted for publication.

## Funding

SJ is the recipient of an Alberta Innovates Health Solutions undergraduate studentship (2020).

## Conflict of Interest

The authors declare that the research was conducted in the absence of any commercial or financial relationships that could be construed as a potential conflict of interest.

## Publisher's Note

All claims expressed in this article are solely those of the authors and do not necessarily represent those of their affiliated organizations, or those of the publisher, the editors and the reviewers. Any product that may be evaluated in this article, or claim that may be made by its manufacturer, is not guaranteed or endorsed by the publisher.

## Abbreviations

CMV: cytomegalovirus
CVB3: Coxsackievirus B3
E7: echovirus-7
EBV: Epstein-Barr virus
EVs: enteroviruses
EV-A71: enterovirus 71
EV-D68: enterovirus 68
Hanta: Hantavirus
HAV: hepatitis A virus
HBV: hepatitis B virus
HEV: hepatitis E virus
HERV: human endogenous retrovirus
HIV: human immunodeficiency virus
SARS-CoV-2: Severe acute respiratory syndrome coronavirus 2
TOSV: Toscana virus
VZV: varicella zoster virus
WNV: West Nile virus
ZIKV: Zika virus.
